# The Male Fetal Biomarker INSL3 Reveals Substantial Hormone Exchange between Fetuses in Early Pig Gestation

**DOI:** 10.1371/journal.pone.0152689

**Published:** 2016-03-31

**Authors:** Andreas Vernunft, Richard Ivell, Kee Heng, Ravinder Anand-Ivell

**Affiliations:** 1 FBN Leibniz Institute for Farm Animal Biology, 18196 Dummerstorf, Germany; 2 School of Biosciences, University of Nottingham, Sutton Bonington, LE12 5RD, United Kingdom; University of Tasmania, AUSTRALIA

## Abstract

The peptide hormone INSL3 is uniquely produced by the fetal testis to promote the transabdominal phase of testicular descent. Because it is fetal sex specific, and is present in only very low amounts in the maternal circulation, INSL3 acts as an ideal biomarker with which to monitor the movement of fetal hormones within the pregnant uterus of a polytocous species, the pig. INSL3 production by the fetal testis begins at around GD30. At GD45 of the ca. 114 day gestation, a time at which testicular descent is promoted, INSL3 evidently moves from male to female allantoic compartments, presumably impacting also on the female fetal circulation. At later time-points (GD63, GD92) there is less inter-fetal transfer, although there still appears to be significant INSL3, presumably of male origin, in the plasma of female fetuses. This study thus provides evidence for substantial transfer of a peptide hormone between fetuses, and probably also across the placenta, emphasizing the vulnerability of the fetus to extrinsic hormonal influences within the uterus.

## Introduction

INSL3 (Insulin-like peptide 3) is a member of the relaxin family of peptide hormones and is produced in male mammals in large amounts uniquely by the Leydig cells of the mature testes as well as of the fetal testes [1,2]. Whilst its function in the adult mammal is less clear, genetic studies in humans and mice [3–5] show that loss of function of the genes for either the hormone or its receptor, called RXFP2 (relaxin-family peptide receptor 2), leads to cryptorchidism. Fetal Leydig cells produce large amounts of INSL3 shortly after sex determination and the initial differentiation of the testis from the uncommitted gonad. This INSL3 is secreted by the fetal testis and interacts with RXFP2 receptors on the gubernacular ligament linking the ventral surface of the testis to the abdominal wall in the inguinal region [6]. Studies on rats and mice show that INSL3 causes the gubernacular bulb to swell thereby anchoring the testes to the inguinal region at a time of rapid fetal growth when the remaining organs of the abdominal cavity are growing away in an anterodorsal direction. This results in what is referred to as the first transabdominal phase of testis descent [7]. In the female fetus little or no INSL3 is expressed, making this hormone a unique fetal-gender specific marker for gonadogenesis and fetal well-being, particularly during the so-called window of sensitivity for testis development in the first and early second trimester (human, ruminants) or in the last third (rodents) of gestation [8]. No other peptide or hormone offers this exclusive biomarker characteristic for the male fetus. Reflecting these properties, INSL3 has also been shown to be an important fetal biomarker for assessing the impact of environmental endocrine disruption on gestational development [8].

INSL3 was originally identified (under the name Leydig Insulin-like factor, Ley-I-L) as a testis-specific transcript in the adult boar testis [9], and later shown to be secreted as a peptide hormone in this species into both circulating blood as well as into the seminal compartment [10], confirming what had earlier been shown for the rat [11]. Importantly, it was observed that INSL3 was probably being expressed not only as a ca. 6kD A-B heterodimer, as in humans, but probably and to a larger extent also as the ca. 12kD uncleaved A-B-C pro-form [12], which would appear to retain full bioactivity at its cognate receptor [12]. No information, however, is available concerning its expression by the fetal pig testis. Moreover, whilst we have some information about fetal expression in monotocous species, such as the human and bovine, information about expression and distribution of fetal INSL3 in a polytocous species is largely lacking. Where information is available, this is for species such as the rat [8,13,14], where gonadogenesis occurs late in gestation, and thus does not offer a good comparison to those species where gonadogenesis and fetal INSL3 production occur earlier in pregnancy, when other fetal development is still quite immature.

The present study was undertaken because the pig, being polytocous and relatively large, offers several advantages as a model species. We had previously shown that in mid-gestation male fetal INSL3 in the bovine is able to cross the maternal-fetal barrier to become measurable in the maternal circulation [15]. Unlike other hormones, such as the gonadal steroids, which are produced both by the testes as well as by the adrenal glands, INSL3 appears to be uniquely generated by the fetal testes and is thus sex-specific, allowing therefore the opportunity not only to assess fetal-maternal transport, but also inter-fetal transport between male and female fetuses in a polytocous species. There have been suggestions in the literature that certain intersex conditions amongst pig litters are more frequent than in other species [16,17], possibly implying some kind of freemartinism or other inter-fetal hormonal influence. Moreover, disruption of testicular descent in pigs is frequently observed (0.2–2.0%)[18]. There is some evidence for inter-fetal transfer of small steroid hormones such as testosterone [19,20] in polytocous species, but the present study is the first to show evidence of inter-fetal transport for a larger peptide hormone. The primary objective of this study was therefore to make use of a large polytocous species in order to investigate and quantify the expression of the peptide hormone INSL3 in different fetal compartments across gestation, thus shedding light on this hormone’s potential to cross significant fetal barriers with relevance for disruption of sexual development. A secondary objective addressed the expression of INSL3 in the maternal circulation in both conceptive and non-conceptive cycles.

In the present study we have collected comprehensive fetal fluid samples from early, mid and late gestation from Landrace pigs, including corresponding maternal serum, also from the non-pregnant animals. By measuring INSL3 in these samples, we can show that there is substantial inter-fetal transport of this male-specific hormone within the allantoic compartments in early gestation (GD45), though less so at later time-points, nor for other fetal fluid compartments. It therefore seems likely that at least in early gestation during the sensitive window for gonadogenesis there is some hormonal exchange between neighbouring fetuses, which may impact on subsequent sexual development.

## Materials and Methods

### Animals

Pubertal German Landrace gilts were housed, sampled and slaughtered within the experimental facilities of the Leibniz Institute for Farm Animal Biology (Dummerstorf, Germany). The entire study and all procedures involving animal handling and treatment were approved by the Leibniz Institute Animal Welfare Officer, and by the state Committee for Animal Use and Care of the Agricultural Ministerial Department of Mecklenburg-Vorpommern, Germany (Permit no. LALLF MV/TSD/7221.2–2.1-009/2 and LALLF MV/TSD/7221.3–1.1-015/09). Oestrus was synchronized in all gilts by 15-day long feeding of altrenogest (16 mg altrenogest/day/ gilt; Regumate, Janssen-Cilag GmbH, Neuss, Germany). Twenty-four hours after the last altrenogest, each animal received a single intramuscular injection of 850 IU equine chorionic gonadotropin (eCG; Pregmagon, IDT Biologika, Dessau-Tornau, Germany). Ovulation was induced 80 h later by administration of 50 micrograms GnRH agonist (Gonavet Veyx, Veyx-Pharma GmbH, Schwarzenborn, Germany). Artificial inseminations were performed 24h and 38h after GnRH administration except in control animals (not pregnant). Blood samples from live animals were collected using standard veterinary diagnostic procedures. Animals were euthanized in an EU approved research slaughterhouse following standard slaughter procedures with electric stunning and exsanguination. Data from 58 gilts aged nine months and with a body weight of ca. 140 kg were included in the study.

### Plasma sampling in Sows

For the measurement of INSL3 in sows during the cycle and pregnancy, blood samples were taken from 24 gilts by puncture of the v. jugularis on days -4, 0, 7, 15, 18 and 28 (day 0 = day of first insemination). Samples were obtained from 11 pregnant gilts (out of 12 inseminated gilts, pregnancy control via B-mode ultrasound examination from the flank on day 28) and 12 non-inseminated gilts (non-pregnant group). From the pregnant gilts, blood samples were also taken at their later slaughtering on days GD45 (n = 4), GD63 (n = 4) and GD92 (n = 3). Blood was collected into tubes containing 1.6 mg EDTA-K /ml (S-Monovette EDTA 9ml, Sarstedt, Nuembrecht, Germany) and immediately stored on ice. Samples were centrifuged directly after collection and 2 ml plasma stored in Eppendorf tubes at -20C until analysis. Additionally, plasma samples of pregnant (n = 14) and non-pregnant (n = 6) gilts from a separate group of pigs were collected on day 30. For comparison of intact and castrated gilts, plasma samples from another study were available from 12 animals, synchronized and inseminated as above, which underwent ovary-hysterectomy on GD15 and were slaughtered 11–13 days thereafter.

### Samples from fetuses

Pregnant gilts were slaughtered on GD30 (n = 16; 197 fetuses), GD45 (n = 4; 70 fetuses), GD63 (n = 4; 62 fetuses) and GD92 (n = 3; 51 fetuses). Amniotic fluid could be collected on GD30, GD45, GD63 and GD92, allantoic fluid on GD45, GD63 and GD92, and fetal blood on GD63 and GD92. Additionally, fetal tissues were collected on GD30 (for only 92 fetuses) and GD45 for gender analysis by PCR (see below). On GD63 and GD92, gender was determined visually. On isolating the uterus at slaughter, the position of each fetus was recorded and the fetus fixed by hand. Then the uterus and the allantoic sac were opened with a scalpel (except on GD30) and 2ml allantoic fluid carefully aspirated using a syringe. Then the amnion sac was punctured with a cannula and 2ml amniotic fluid aspirated. For fetuses on GD63 and GD92, after opening the amnion sac, the umbilical vein was punctured with a cannula and at least 2ml fetal blood collected into plasma tubes, as above, stored on ice, centrifuged, and aliquots frozen at -80°C until analysis. A comparison of INSL3 concentrations comparing plasma collected from umbilical vein, umbilical artery, and direct heart puncture at GD92 (N = 10) showed no significant differences. Great care was taken at all times to avoid any possible cross-contamination of fluid samples.

**Fig 1:**
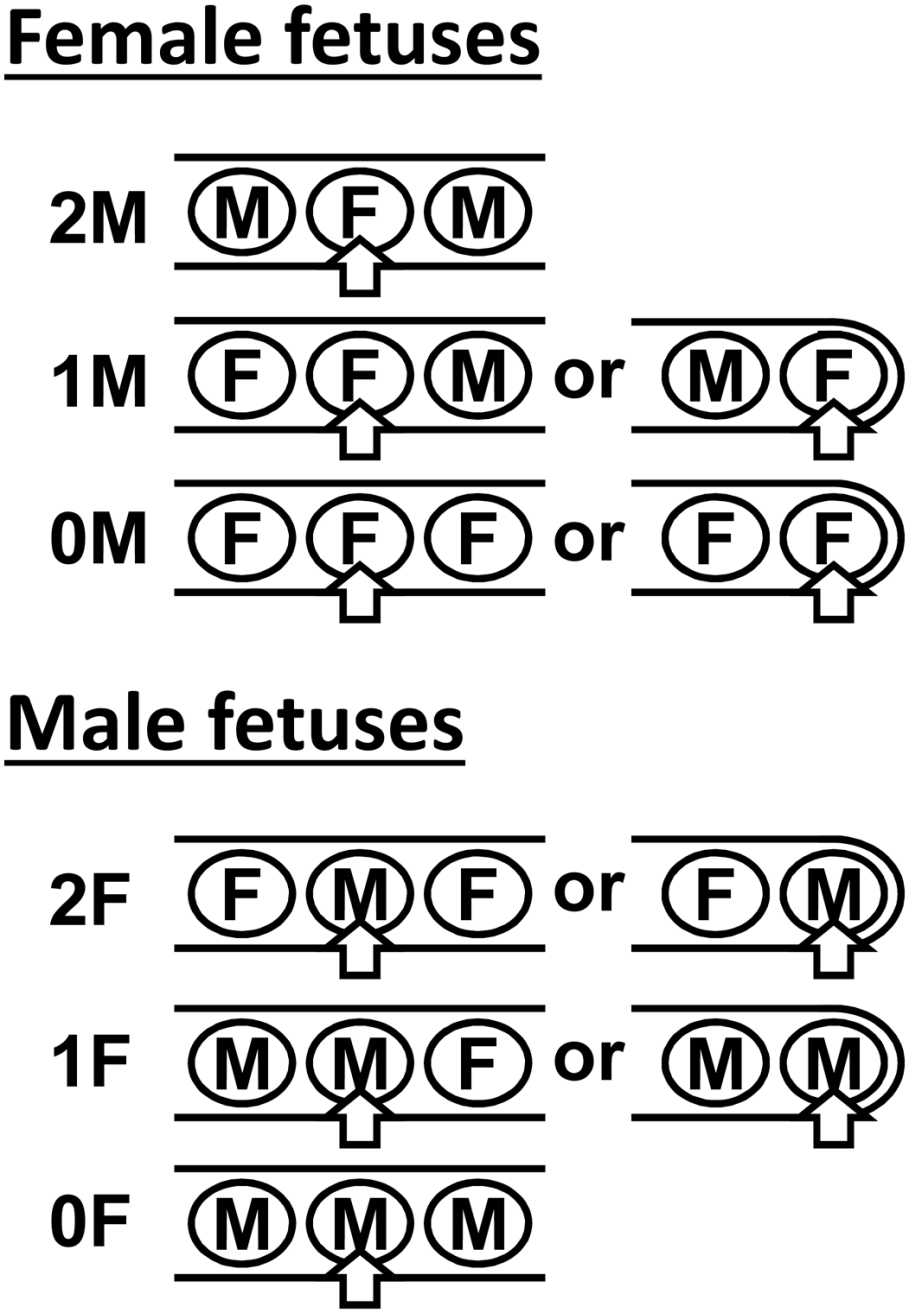
Diagram to indicate scoring (2M, 1M, 0M, 2F, 1F, 0F) of fetal position for fetuses within (left column) or at the fundal end (right column) of a uterine horn. Fetuses at the cervical end of a uterine horn, and thus in contact with the terminal fetus from the other horn, were scored using the left column.

Particular care was taken to note the positions of all fetuses within the uteri at sample collection (Fig 1). Essentially following the classification of Wise & Christenson [21], male fetal samples were labelled 2F when a male fetus was located between two female fetuses or at the fundal end of a uterine horn; 1F when a male fetus was adjacent to one other male fetus; and 0F when located between two other male fetuses within a uterine horn. For female fetuses, these were labelled 2M when located between two male fetuses within a uterine horn; 1M, when adjacent to a single male fetus either within a uterine horn or at its fundal end; and 0M, when a female fetus has no direct contact to a male fetus. At the cervical end of each horn, where the uterus bifurcates, the last fetuses in left and right horns are in direct contact, similar to those within a uterine horn, and were accordingly labeled.

### Immunoassays

Pig INSL3 was measured using a modified version of a time-resolved fluorescence immunoassay (TRFIA) previously developed and validated for ruminants [15]. Being both ungulate, the sequence of the bovine A-B heterodimer against which the antibodies had been raised was highly homologous (94%) to the porcine sequence [22]. Validation controls showed that the heterologous assay indicated no cross-reaction with porcine relaxin, insulin, IGF1 or IGF2, with a limit of detection (LOD) of 20 pg/ml bovine INSL3, and intra- and inter-plate coefficients of variation <2% and <10%, respectively, across the assay range (20pg/ml to 6ng/ml). Samples of castrated bull serum, gilt serum, mid-gestation female porcine allantoic fluid, and mid-gestation female amniotic fluid, each spiked with 0.5 ng pure bovine INSL3, indicated recoveries of respectively 98.4%, 93.5%, 101.9%, and 114.3%. Parallelism was confirmed using serial dilutions of bull serum, boar serum, and mid-gestation male porcine amniotic fluid, all with similar resulting slopes (not shown). There was insufficient male porcine allantoic fluid with high endogenous INSL3 to include this matrix in the comparison.

In order to monitor conceptive and non-conceptive estrous cycles, luteal function was assessed by progesterone, using a well-established immunoassay previously validated in pigs [23].

### Determination of fetal sex

Fetal sex was determined by specific PCR from genomic DNA isolated from muscle tissue of GD30 and GD45 fetuses, essentially following the method of Sembon et al. [24]. Whereas tissue was available for all the GD45 fetuses, tissue from only 92 of the 197 fetuses collected at GD30 could be collected. Frozen tissue was incubated at 55C for 3 hours in digestion buffer (10mM Tris base, 75mM NaCl, 25mM EDTA, 1% (w/v) sodium dodecyl sulphate, 0.1mg/ml proteinase K), then centrifuged at 5000 g and room temperature for 5 mins. DNA was precipitated from the supernatant by an equal volume of isopropanol and washed once with 70% ethanol. The dried genomic DNA pellet was taken up in nuclease-free water and quantitated using an Eppendorf Biophotometer. Degenerate PCR primers (AMEL Forward, 5’-CRCMTTCATTGAYAATTCAC-3’; AMEL Reverse, 5’-CCAGAGGTTGTAACCTTACAG-3’; R = A or G; M = A or C; Y = C or T) were used to amplify a portion of intron 2 of the porcine amelogenin (AMEL) gene that was expected to yield amplicons of differing sizes between the alleles on the X (AMEL-X) and Y (AMEL-Y) chromosomes. PCR was performed using a Rotor-Gene RG-3000 (Corbett Research/Qiagen, VIC, Australia) real-time thermocycler using 100ng of genomic DNA and 2.5pmol/μl of each primer per reaction. After an initial denaturation at 95C for 3 minutes, cycling (denaturation 95C, annealing 61C, elongation 72C; each for 30 secs) progressed for 40 cycles with data capture at the end of each elongation cycle. After a 2 minute final incubation at 40C, melt curves were obtained to determine reaction efficiency and effectiveness. All PCR products were run on 1.5% agarose gels. A single band of 520bp was observed for female fetuses while 2 bands of 520bp and 350bp were seen for male fetuses [24].

### Statistics

For each gestational age the fetuses from 3 to 4 pregnant gilts derived from an inbred population were compared (total fetuses used for analysis: GD45: 38 female, 26 male; GD63: 30 female, 29 male; GD92: 19 female, 31 male; note that some fluids from a small number of fetuses could not be collected). Comparisons for all parameters between sows at any gestational age showed no differences for any parameter based on maternal identity. Consequently, all fetuses at each gestational age were pooled for subsequent analyses. All comparisons were analysed by ANOVA followed by Neumann-Keuls post hoc tests, using the GraphPad Prism v6.0 software. Differences were considered significant at p<0.05.

## Results

### INSL3 in serum from non-pregnant and pregnant sows

**Fig 2:**
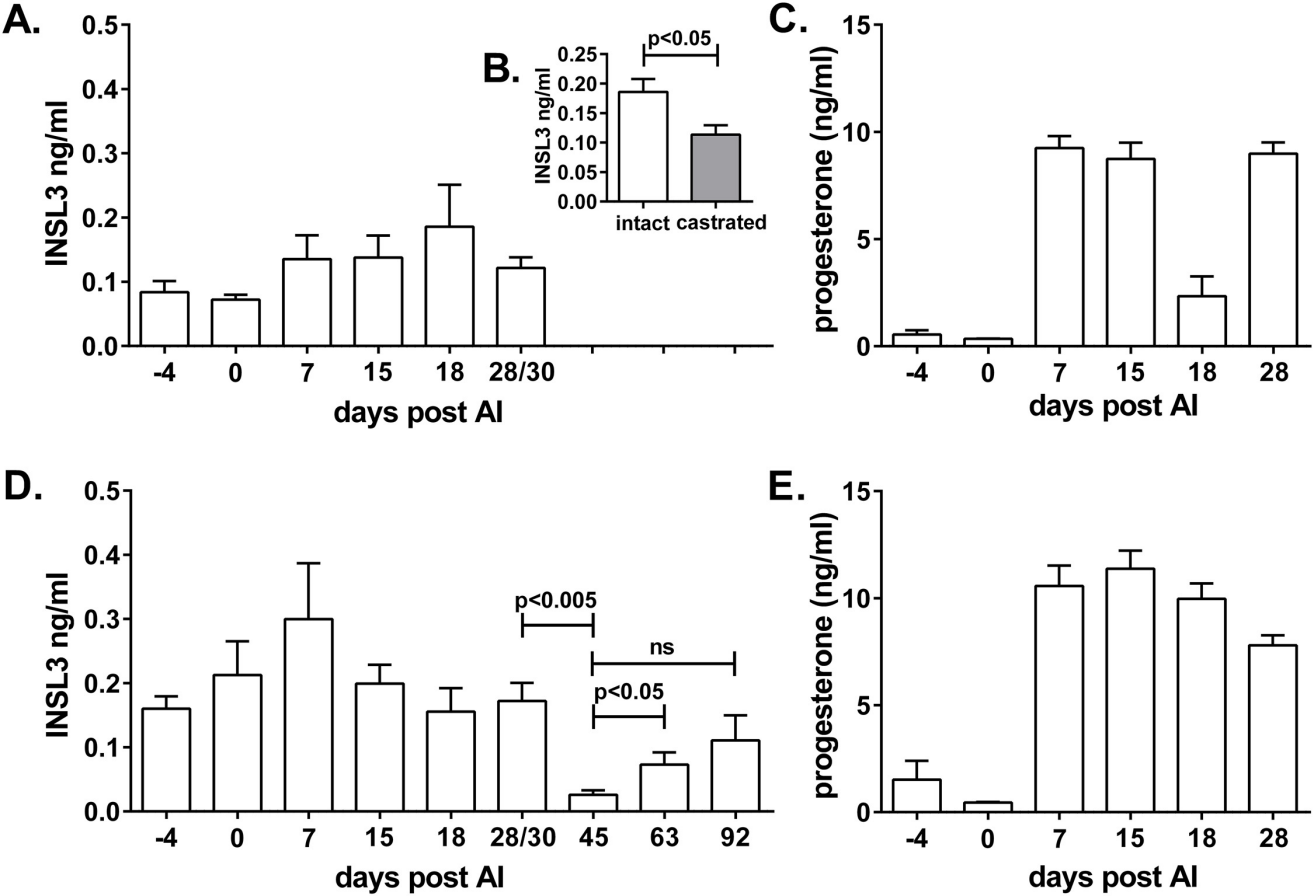
Circulating INSL3 (A,B,D) or progesterone (C,E) in conceptive (D,E) (n = 11) and non-conceptive (A,C) (n = 12) cycles of gilts subjected to hormonal synchronization and artificial insemination (AI) or not. Panel D also includes INSL3 values from later in pregnancy (n = 4–5). Panel B indicates maternal plasma values for pregnant gilts (n = 12) on day GD15 (intact) and following ovariohysterectomy that day with blood sampling 11–13 days later (castrated).

Measuring circulating INSL3 across conceptive and non-conceptive cycles (Fig 2A and 2D) shows neither significant differences nor temporal trends, unlike progesterone which markedly reflects the formation, growth and demise of multiple corpora lutea (Fig 2C and 2E). Comparing circulating maternal INSL3 measured across gestation also indicates no significant temporal changes (Fig 2A and 2D), all values being relatively low. As a control for the source of INSL3 in the female pig, circulating INSL3 was also measured in pregnant sows on GD15 and 11–13 days later after ovariohysterectomy (Fig 2B). The castrated values were significantly lower than in the intact animals.

### INSL3 in different fetal compartments across gestation

**Fig 3:**
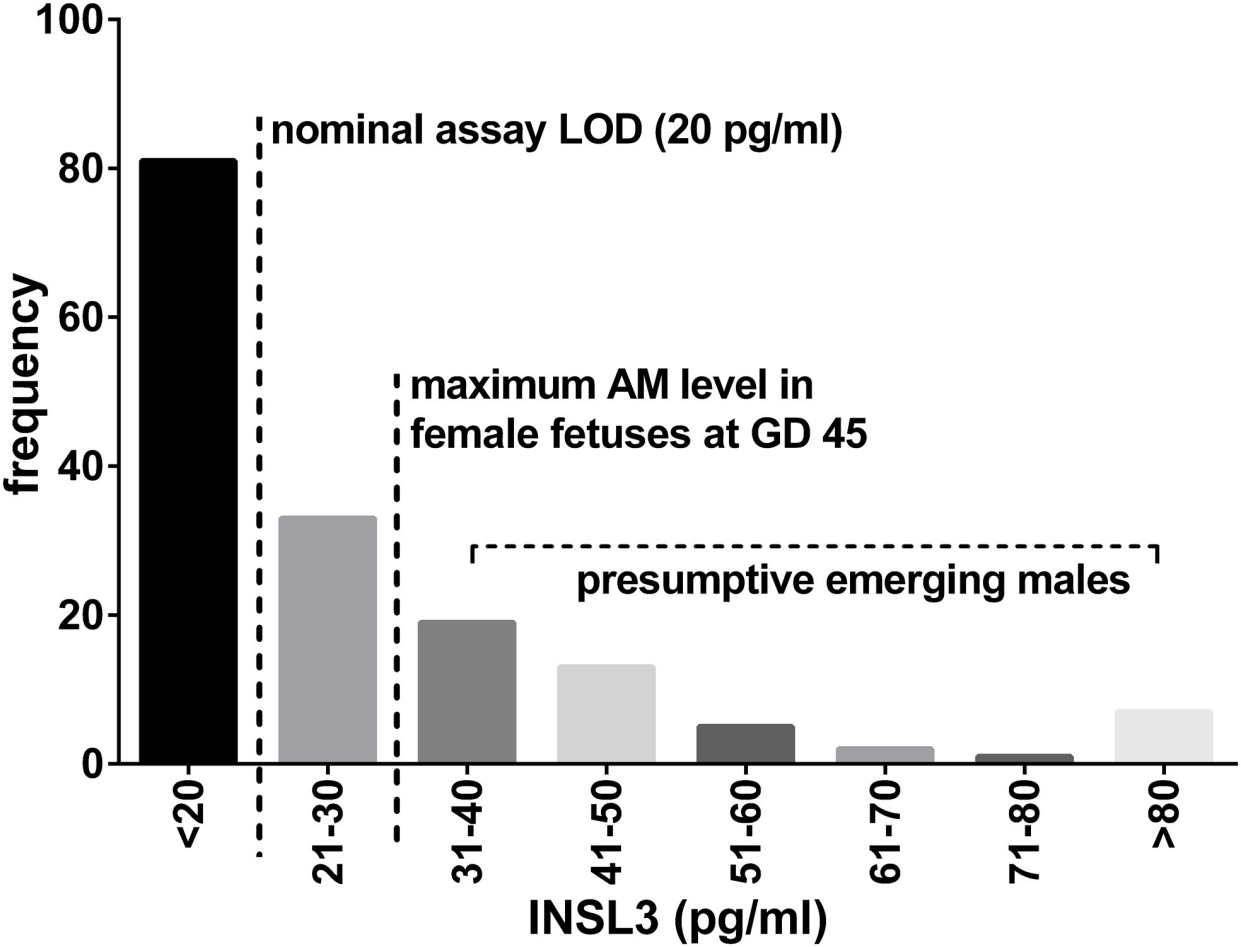
Frequency histogram for INSL3 concentration in amniotic fluid (AM) from all GD30 fetuses, irrespective of fetal sex. LOD, limit of assay detection.

INSL3 was measured in allantoic fluid (AL) and amniotic fluid (AM) from male and female fetuses at GD45, GD63, and GD92 (term is at approximately GD114), and at GD30 in amniotic fluid only. Additionally, INSL3 was measured in fetal plasma at GD63 and GD92. GD30 represents a time-point when sex determination as measured by the expression of sex-specific gonadal genes has just begun [25]. It is consequently not surprising that the expression of INSL3 in amniotic fluid at this time-point is largely below the detection limit (Fig 3), and hence while mean values differ between fetal sex for those fetuses where this could be determined (male, 30.5 ± 26.4 pg/ml; female, 22.7 ± 8.6 pg/ml; p = 0.059) this difference does not reach significance. Whereas 45% of fetuses with INSL3 <20pg/ml were male, this proportion increases to 74% for INSL3 values >20pg/ml, and 100% for values >50pg/ml. Interestingly, of the 4 female fetuses registering INSL3 values >30pg/ml, 3 were located adjacent to at least one male fetus.

**Fig 4:**
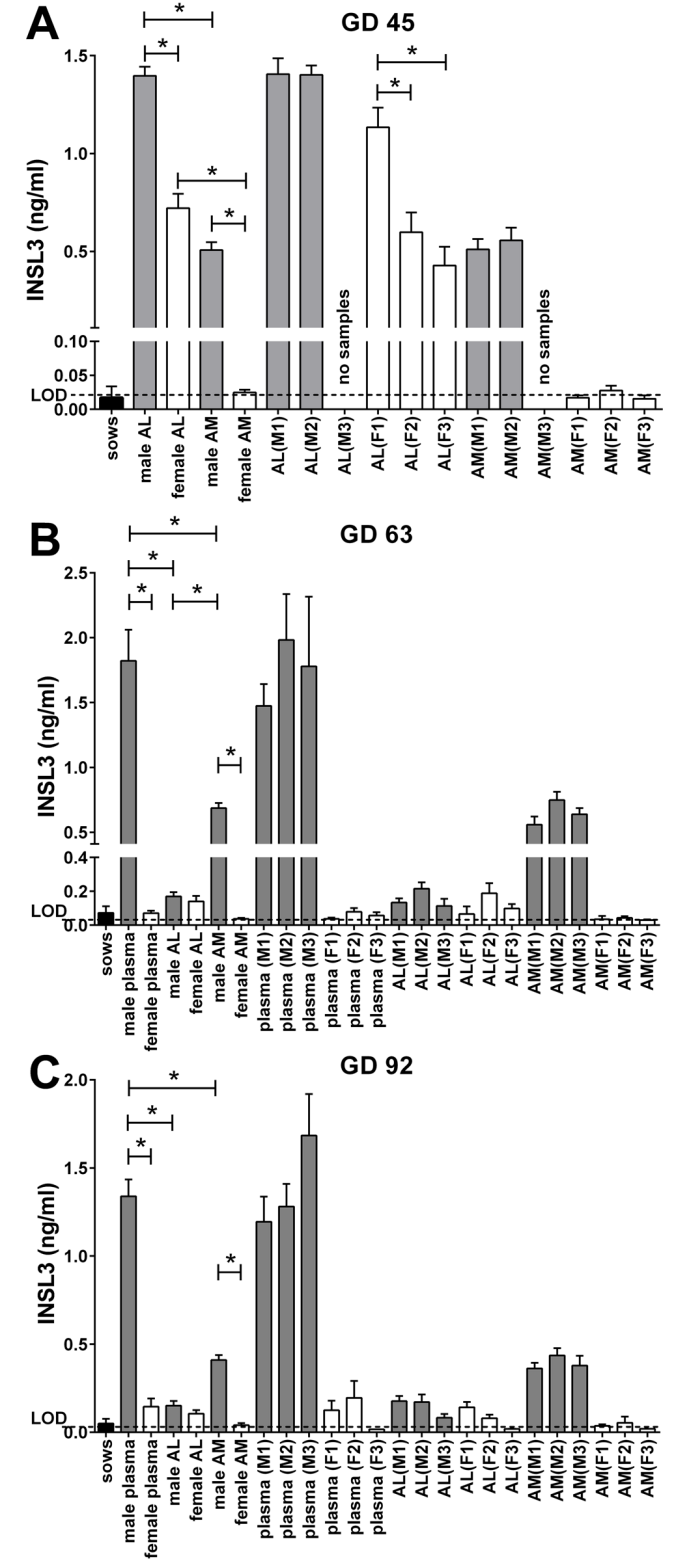
INSL3 concentrations in maternal plasma (sows) and different fetal compartments (plasma; AL, allantoic fluid; AM, amniotic fluid) at (A) GD 45, (B) GD 63, and (C) GD 92, grouped by fetal sex, and/or sorted by fetal position (as indicated in Fig 1). Numbers in italics below histograms indicate respective n values. * indicates significant difference p<0.05. LOD, limit of assay detection.

GD45 marks the phase of transabdominal testicular descent [26]. Both allantoic fluid INSL3 (AL) as well as amniotic fluid INSL3 (AM) concentrations differ significantly between fetal gender (Fig 4A), with INSL3 concentration in male allantoic fluid (ca. 1.4 ng/ml) being approximately three times that for amniotic fluid (ca. 0.5 ng/ml). However, whereas for female amniotic fluid INSL3 concentration is generally below the limit of detection, this is not the case for female allantoic fluid, where quite substantial mean INSL3 concentration was recorded. The only known potential source for such INSL3 is the male fetus. Thus when INSL3 concentrations in female allantoic fluid (AL) are plotted against fetal position (Fig 4A), higher INSL3 values are determined for female fetuses adjacent to one (1M) or two (2M) male fetuses, compared to those not adjacent to a male fetus (0M). No such position effects are noted for male fetuses, nor for male and female fetuses when assessing amniotic fluid (AM; Fig 4A).

In mid-gestation at GD63 all samples from female fetuses were close to or at the limit of detection (Fig 4B). In contrast, male fetal plasma indicated a circulating concentration of ca. 1.8 ng/ml, amniotic fluid (AM) a concentration of ca. 0.7 ng/ml, and allantoic fluid (AL) ca. 0.2 ng/ml (Fig 4B). For none of these body fluids, and in contrast to GD45, was a relationship to fetal position detected. Nor was there any significant sex difference for the allantoic fluid samples, unlike for plasma or for amniotic fluid.

This situation does not change markedly at GD92 (Fig 4C). INSL3 concentrations in male plasma (ca. 1.4 ng/ml) and male amniotic fluid (ca. 0.4 ng/ml) are significantly greater than for female fetuses, whereas male allantoic fluid INSL3 concentration is substantially lower (ca. 0.15 ng/ml) and not different from female allantoic fluid (Fig 4C).

For GD92 there appears not to be an impact of fetal position on plasma, allantoic or amniotic fluid concentrations of INSL3 for female fetuses (Fig 4C). Nevertheless, other than for 0M samples of female plasma, amniotic or allantoic fluid, where female fetuses have no direct contact to a male fetus, and which consequently show INSL3 values at the assay limit of detection (20 pg/ml), the remaining samples of plasma and allantoic fluid, though not amniotic fluid, all derived from female fetuses contacting one or more male fetus, indicate INSL3 concentrations significantly (p<0.05) above the detection limit. At GD63 (Fig 4B), most female plasma and allantoic fluid samples are significantly (p<0.05) above the limit of detection, the latter in fact being little different from male allantoic fluid samples at that stage.

### Maternal INSL3 concentrations during gestation

Early in gestation (≤ GD30) there are no differences evident for INSL3 in maternal plasma between pregnant and non-pregnant sows (Fig 2D). However, at GD45 maternal circulating INSL3 significantly falls to the level of assay detection. This is not the case at GD63 and GD92, when maternal INSL3 is significantly above the GD45 level (Fig 2D).

## Discussion

The peptide hormone INSL3 has proved itself very useful as a constitutive testis-specific biomarker for the assessment of pubertal development in several species [11, 27–29], as well as in male aging [30]. Whilst predominantly a male-specific hormone, two recent studies have highlighted its importance as a biomarker also of antral follicle development in cows [31] and women [32]. Although circulating concentrations in these species are low, it is apparent that INSL3 secretion reflects the emergence and growth of a wave of antral follicles and that such secretion ceases following the LH surge. The data presented in Fig 2 support such a notion also for the female pig, with higher values in mid-cycle, presumably reflecting the large number of emerging follicles [33,34], and no significant differences between conceptive and non-conceptive cycles. Unlike progesterone, there appears to be little contribution to circulating INSL3 from luteal tissue. Whilst removal of the ovaries leads to a significant drop in circulating INSL3 (Fig 2B), the still high residual suggests that there might be alternative sources of this hormone in female pigs, though there is no other evidence to support this. The very low values for circulating maternal INSL3 in mid-gestation (GD45; Fig 2D), compared to earlier and later time-points, are also interesting. This might imply that at least in the second-half of gestation some of the maternal INSL3 could be derived from male fetuses in utero, as previously shown for the cow [15], and that it is unlikely that there is any INSL3 transport in the other direction.

The main purpose of this study was to investigate the expression and distribution of INSL3 derived from the fetal testis across gestation in a polytocous species. Firstly, the results fully support the view derived from other species that INSL3 is uniquely derived from the Leydig cells of the fetal testes, beginning expression at about GD30 or shortly thereafter, leading to a maximum in the fetal circulation in mid gestation at about GD63, and declining slightly in later pregnancy. This trajectory is in full agreement with its proposed role to promote the initial transabdominal phase of testicular descent which in pigs begins at around GD40 [26]. Retrospectively, it seems very likely that INSL3 is the low molecular weight peptide derived from the fetal porcine testis earlier predicted by Fentener van Vlissingen et al. [35] and Visser & Heyns [36].

This study was able to assess INSL3 concentrations in three fetal compartments: plasma, amniotic fluid and allantoic fluid, whereby plasma could only be collected from later gestational time-points. Early in pregnancy (GD30), there is a very small amount of amniotic fluid and substantial volumes of allantoic fluid [37]. At this time the fetal skin is not keratinized and amniotic fluid is very much a fetal exudate with contributions from the placenta. At GD45 amniotic fluid is increasing in volume, though still substantially less than the volume of allantoic fluid, whereas by mid-gestation at GD63 amniotic fluid volume has increased to ca. 150 ml, and allantoic fluid to a maximum of almost 300 ml [37]. By this time, allantoic fluid has become quite dilute and comprises very much a maternal product of the epitheliochorial placenta [38,39]. By the end of gestation amniotic fluid and allantoic fluid volumes are both reduced, each being about 100 ml, presumably due to the space limitations of having a large litter.

Looking at INSL3 in these different compartments of the male fetus, then it is evident that whereas the amniotic fluid concentration remains relatively stable throughout gestation, being consistently about one-third that of fetal plasma, allantoic fluid is changing quite dramatically, from having very high values early in gestation, to having only very low concentrations later in gestation. This change is consistent with its changing role, from being a continuum between fetus and placenta early in gestation prior to fetal keratinization, to being a specialist hypo-osmotic nutrient environment largely though not exclusively contributed by the mother in the second half of pregnancy [38,39].

A very exciting result from this study is the observation that early in gestation (GD45, and possibly GD30) there appears to be substantial hormonal exchange between male and female fetuses, though less at later time-points. The observation that some samples of female plasma and allantoic fluid, though not of amniotic fluid, later in gestation were still above the limit of assay detection for INSL3 suggests that hormonal exchange may still be occurring in the second half of gestation, though it cannot be excluded that there may be a small undescribed source of INSL3 in the female fetus. However, we consider this alternative unlikely since plasma from female fetuses which are surrounded by other females in utero consistently indicates INSL3 concentrations at or below the assay limit of detection. Because of the fetal gender-specific property of INSL3, a clear transport of hormone from male to female fetuses is apparent at GD45, and then more or less exclusively within the allantoic compartment. It has been suggested that in pigs adjacent conceptuses do not share blood supplies, and hence cannot exchange male-determining factors, thus precluding the possibility for freemartinism, common in predominantly monotocous species [40]. However, these results suggest that this supposition is not entirely valid, and that indeed some hormones can be exchanged. This is significant because pigs have been shown to have a relatively high incidence of intersex phenotypes, predominately in female (XX) pigs [16,17,41], which would appear to have their origin early in gestation [17]. Although genetic causes are likely, the present study suggests that inter-fetal hormonal exchange may also be a relevant factor. This was suggested by an earlier study on the impact of fetal position on male pig phenotype [42], where a significant effect of being located *in utero* between two other male fetuses on weight gain under restricted feeding conditions was observed. vom Saal and colleagues using an interventional paradigm in rodents had also indicated a transfer of testosterone between fetuses by diffusion across adjacent amniotic membranes [19,20]. Moreover, it has been shown that steroidogenesis and its metabolism in adult rats and mice also appears to depend on previous fetal position [43,44]. Similarly, measurement of anogenital distance (AGD), an androgen-dependent phenotypic parameter, is greater for female fetuses adjacent to two male fetuses in rats [45], and in pigs AGD in females has been shown to depend on the proportion of males in the litter [46]. The present study differs from these studies in that it relates to a non-interventional situation, earlier in pregnancy, and importantly involves a moderately large peptide hormone rather than smaller, more hydrophobic steroids.

Very little is known about the possible transport mechanisms that might be involved here. Whereas for steroids it is generally assumed that these small and relatively lipophilic molecules may move across membranes by passive diffusion, for larger peptide hormones this is unlikely. In general, in early gestation solutes in amniotic fluid are believed, like water, to follow osmotic gradients between fetal blood and placenta, but involving an active transport mechanism [47]. This is also true for the transport of insulin, which is similar in structure to INSL3, across comparable barrier systems [48]. The nature of the active transport system involved in the inter-fetal transfer of INSL3 is however unknown.

In conclusion, this study has shown in a polytocous species that there is a physiological opportunity for the exchange of peptide hormones between fetuses, at least early in gestation, if not later. This exchange appears to involve the allantois, rather than the amniotic compartment, and may account for above-background levels of the hormone INSL3 in the plasma of female fetuses also later in gestation. Once one accepts the notion that fetal hormones may reach the placenta and possibly the maternal circulation, then it presents the hypotheses not only that endocrine exchange between fetus and mother is possible in both directions, but also that fetal gender may have an endocrine role to play in the etiology of certain maternal pathologies [49]. As a corollary, this study also emphasizes the vulnerability of the mammalian fetus to hormonal influences mediated by the mother, including those with endocrine disrupting properties.
