# Correction: The Male Fetal Biomarker INSL3 Reveals Substantial Hormone Exchange between Fetuses in Early Pig Gestation

**DOI:** 10.1371/journal.pone.0157954

**Published:** 2016-06-15

**Authors:** Andreas Vernunft, Richard Ivell, Kee Heng, Ravinder Anand-Ivell

The following information is missing from the Acknowledgements in the published article: “The publication of this article was funded by the Open Access Fund of the Leibniz Association.”
